# Supplementary material for: Deep learning the structural determinants of protein biochemical properties by comparing structural ensembles with DiffNets
Source: Nat Commun. 2021 May 21;12:3023. doi: 10.1038/s41467-021-23246-1 (PMC8140102; doi:10.1038/s41467-021-23246-1)
Supplement: Supplementary file 1 — Supplementary Information [file 41467_2021_23246_MOESM1_ESM.pdf]

Supporting Information

Deep learning the structural determinants of protein biochemical properties by comparing structural ensembles with DiffNets

Michael D. Ward[1,2], Maxwell I. Zimmerman[1,2], Artur Meller[1,2], Moses Chung[1,2], S.J. Swamidass[3], and Gregory R. Bowman[1,2]*

[1]Department of Biochemistry & Molecular Biophysics, Washington University School of Medicine, St. Louis, MO 63110, USA
[2]Center for the Science and Engineering of Living Systems, Washington University in St. Louis, St. Louis, MO 63110, USA
[3]Department of Pathology & Immunology, Washington University School of Medicine, St. Louis, MO 63110, USA

*To whom correspondence should be sent: g.bowman@wustl.edu

Contents

Expectation maximization algorithm details.

Supplementary Figure 1. Self-supervised DiffNets are robust across a range of expectation maximization bounds.

Supplementary Figure 2. Self-supervised DiffNets improve ability to predict properties of variants outside the training.

Supplementary Figure 3. Impact of expectation maximization on what features a DiffNet uses to distinguish variants.

Supplementary Figure 4. DiffNet analysis suggests conformational changes on switch-II are important for distinguishing high-and low-duty myosin isoforms.

The expectation maximization (EM) algorithm

We hypothesize that it is possible to use EM to learn the association between individual structures and a biochemical property of interest. EM is a statistical method that allows the parameters of a model to be fit, even when the outputs of the model cannot directly be observed in the training data (i.e. when they are hidden)[1]. In our case, the hidden variables are the elements of a vector of numbers, associated with every structure in the simulation training data. Each variable should be a 1 if it is associated with the biochemical property and 0 otherwise, but we do not know what the correct value is, they are hidden. First, this vector is initialized to reasonable starting values. Next, during the Maximization step (M-step) we train a neural network to create a mapping between each structure's descriptors (i.e. XYZ coordinates) and the current estimate of the hidden variables. Then, during the Expectation step (E-step), we re-estimate our hidden variables using the trained model and the region constraints that specify how many structures we expect to be associated with the biochemical property of interest. Finally, we alternate between the E- and M-steps for a predefined number of steps.

Initialization and progression of the algorithm

The EM algorithm alternates between E- and M-steps. To initialize the algorithm, we pick an output vector $Y = (y_i)$ such that all values corresponding to simulation frames of one class of variant are assigned 0s, and all other values are assigned 1s. This is our initial guess for our hidden variables, $K = (k_i)$ (Eq. 1). Each element of K is our current estimate of which structures are associated with the biochemical property of interest. Next, the M-step fits a neural network using K as targets (Eq. 2),

$$K_1 \leftarrow \; Y_{init} \tag{1}$$

$$W_1 \; and \; Y_1 \; \leftarrow M - step \quad (K_1, D), \tag{2}$$

where W_1 is the tuned weights of the neural network and Y_1 is the output of the model using these weights with the data. This output vector, Y_1, is used in the E-step to compute the next guess for the hidden variables K (Eq. 3). The next iteration repeats the E- and M-steps,

$$K_2 \; \leftarrow E - step \quad (Y_1) \tag{3}$$

$$W_2 \; and \; Y_2 \; \leftarrow M - step \quad (K_2, D), \tag{4}$$

Subsequent iterations repeat these steps for a predefined number of steps. As the algorithm progresses, both the K and Y vectors should converge to a value that indicates the extent that a structure is associated with the biochemical property of interest. They should label the structures associated with the property with high probabilities, and the other structures with low probabilities.

Expectation step

The E-step computes the expected values of the hidden variables K from the outputs Y conditioned on constraints defined by the user (e.g. only 0-30% of simulation data is expected to be associated with the property of interest for one class of data, and 40-70% for the other class). The expectation of the hidden variables is the probability-weighted average of all binary

realizations of binomial distributions parameterized by Y that assign the right number of structures as being associated with the property of interest. Conceptually, the expectation is computed by, first, enumerating all binary realizations of Y, each denoted as a vector of boldface variables $\boldsymbol{Y} = (y_i)$. Second, vectors that do not have the right number of structures according to the user-defined constraints are rejected. Third, the remaining vectors are scored by their probability according to Y, and, finally, a probability-weighed average of the binary vectors is computed. This average vector is the expectation, and is assigned to K. A straightforward Python implementation of this calculation can be found here (https://github.com/bowman-lab/diffnets/blob/master/diffnets/exmax.py) under the function name "expectation_range_EXP".

While conceptually clear, computing K in this way is very slow because there are exponentially many realizations of Y that must be enumerated. Fortunately, the expectation is computable in polynomial time. Here, we treat the structure labels as binary random variables following binomial distributions parameterized by Y. For each class of data, the expectation of these variables is assigned to elements of K. Given the user-defined constraints about the number of structures associated with the property of interest, this update can be derived from Baye's Rule,

$$k_s = E[y_s \mid S_L \leq \ y_r \leq S_U \ ] \tag{5}$$

$$= P(y_s \text{ is } 1) * \left(\frac{P(S_L - 1 \leq y_r - y_s \leq S_U - 1}{P(S_L \leq \ y_r \leq S_U)} \right) \tag{6}$$

where y_r is the integer sum of the binary labels associated with the structures of the given class which are associated with the biochemical property of interest, y_s is the binary label of a given structure (site *s*), $P(y_s \text{ is } 1)$ is the probability that the structure is associated with the biochemical property according to Y, the numerator is the probability that the number of structures associated with the biochemical property (ignoring site *s*) ranges from $S_L - 1$ to $S_U - 1$, and the denominator is the probability that the number of structures associated with the biochemical property range from S_L to S_U. S_L and S_U are equal to the number of structures in a given class that are associated with the biochemical property of interest according to the user-defined constraints.

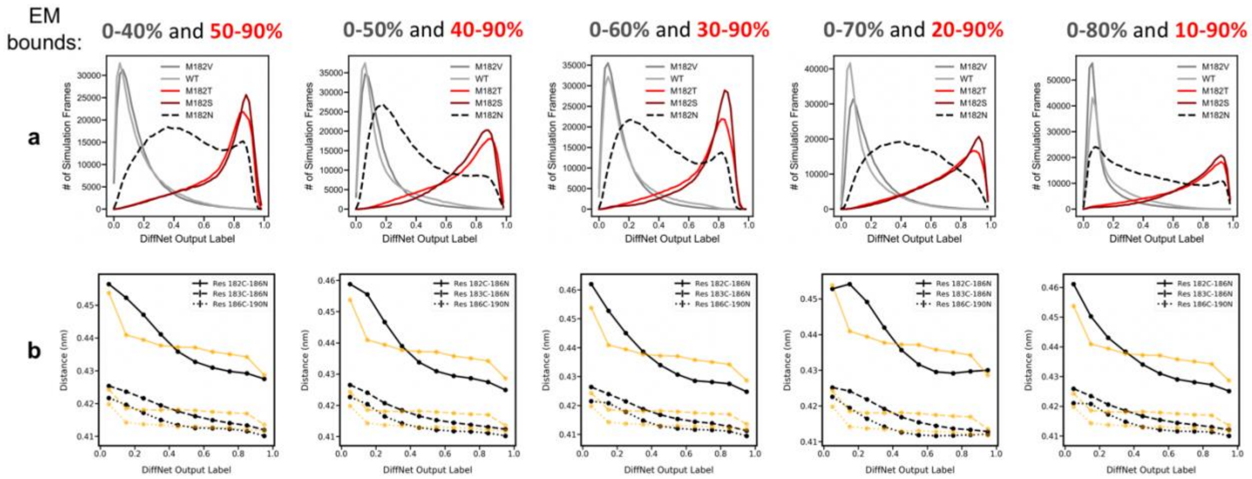

Supplementary Figure 1. Self-supervised DiffNets are robust across a range of expectation maximization bounds. **(a)** Histogram showing DiffNet output labels across all simulation frames from M182T and M182S (red – highly stable variants in training set) versus WT and M182V (grey – less stable variants in training set) across a range of expectation maximization bounds. Predictions on a less stable variant not seen during training (M182N) are also shown (black dotted line). **(b)** Three key hydrogen bond lengths in helix 9 as a function of the DiffNet output label (n=1,300,420 for each plot) (yellow – supervised, black – self-supervised), which ranges from zero for structures associated with low stability to one for structures associated with high stability. The distances are between the carbonyl carbon of the i'th residue and the nitrogen of the (i+4)'th residue. Standard error bars are not visible since the standard error is smaller than scatter points.

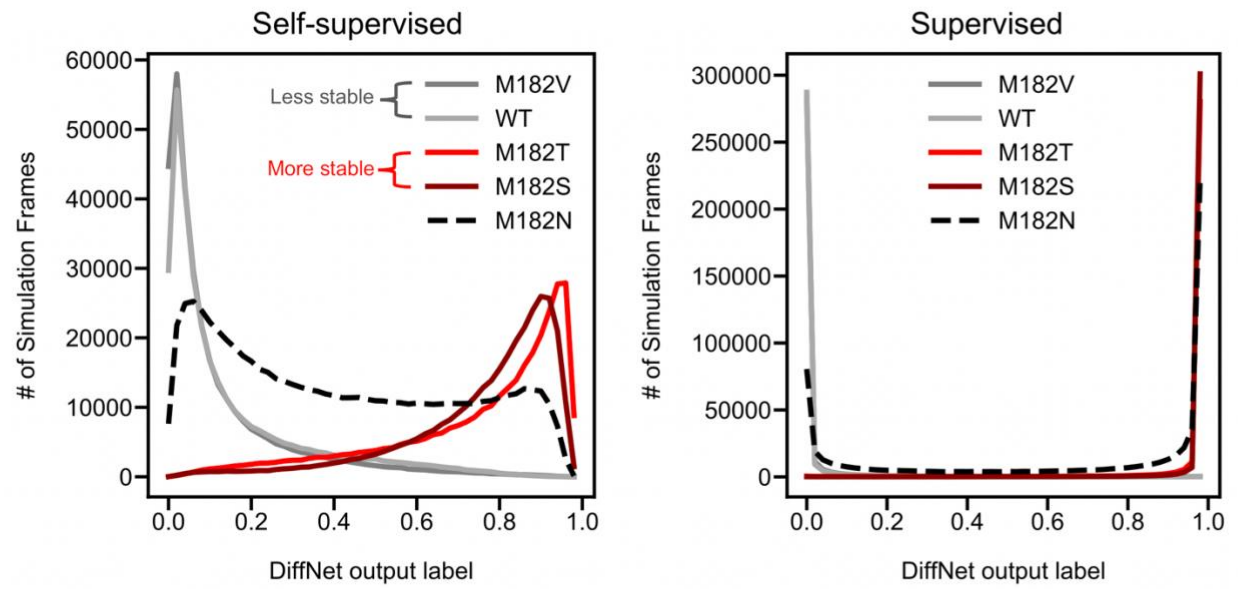

Supplementary Figure 2. Self-supervised DiffNets improve ability to predict property of a variant outside the training. Histogram of final DiffNet output labels for all simulation data points organized by variant (red – more stable variants, grey – less stable variants, black – less stable variant not seen during training) for a self-supervised DiffNet and a supervised DiffNet.

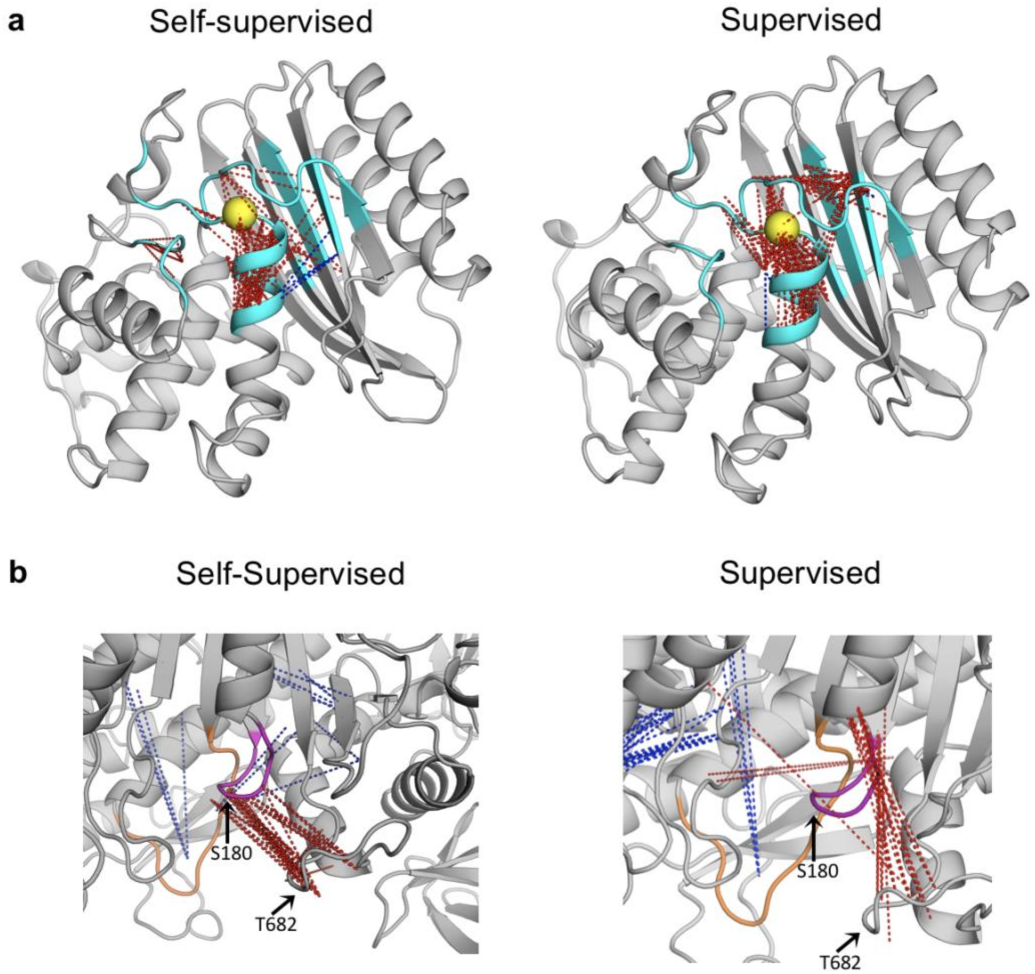

Supplementary Figure 3. Impact of expectation maximization on what features a DiffNet uses to distinguish variants. Dotted lines indicate distances between two atoms that change in a way that is strongly correlated with an increased DiffNet output label. Red indicates the atoms move closer together as the output label increases, blue indicates atoms moving away from each other. Results for β-lactamase variants and myosin are shown in **(a)** and **(b)** respectively. In **(a)**, protein atoms are colored cyan if they are near the mutation, which indicates that they were included in the classification task and considered for the distance correlation calculation. The site of the single point mutation is highlighted with a yellow sphere.

Self-supervised and supervised DiffNets both capture helix 9 compaction as the key feature that distinguishes stability in β-lactamase variants and we observe no qualitative improvement for the self-supervised model. In contrast, a self-supervised DiffNet correctly hones in on the importance of S180 dynamics in determining duty-ratio in myosin isoforms, but a supervised DiffNet does not.

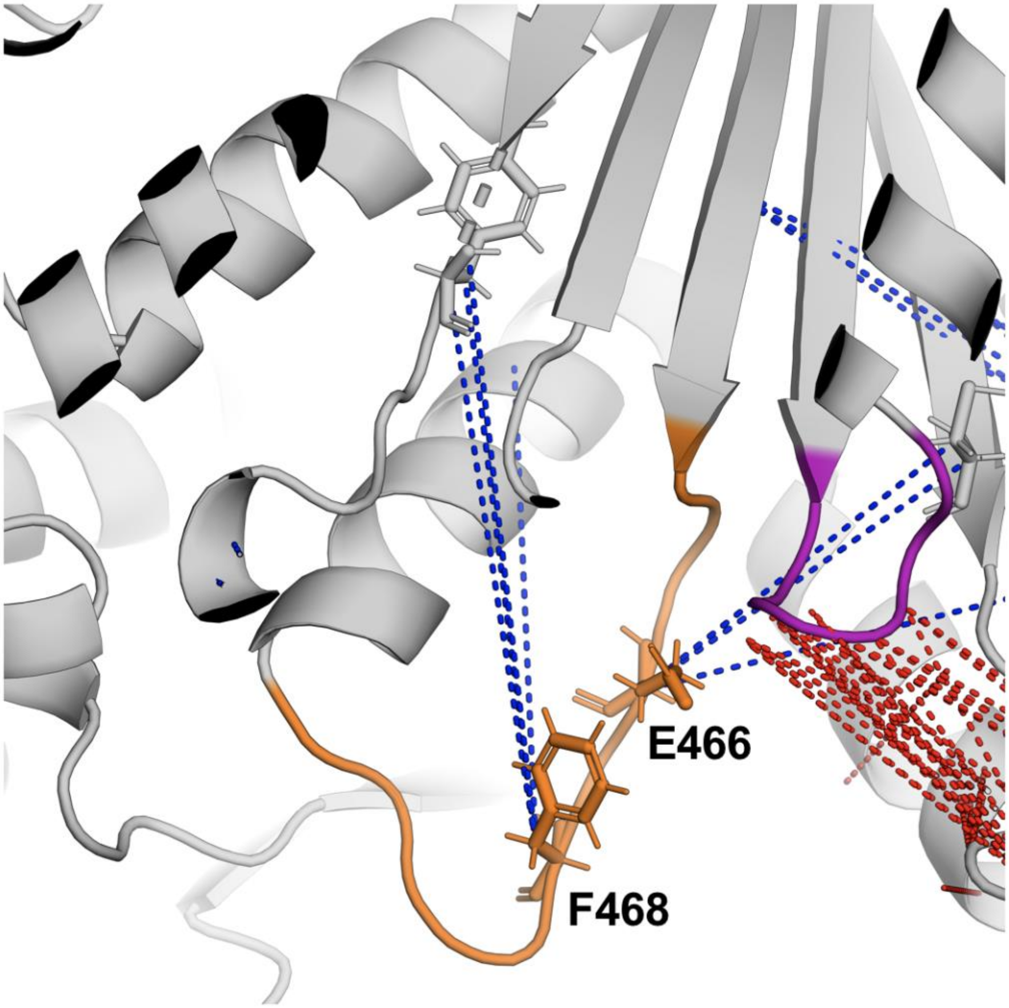

Supplementary Figure 4. DiffNet analysis suggests conformational changes on switch-II are important for distinguishing high-and low-duty myosin isoforms. Dotted lines indicate distances between two atoms that change in a way that is strongly correlated with an increased DiffNet output label. Red indicates the atoms move closer together as the output label increases, blue indicates atoms moving away from each other. Switch-II is colored orange and the p-loop is colored purple.

Self-supervised DiffNet predicts that distance changes involving residues on switch-II (F468, E466) distinguish high and low-duty motor myosins. These residues are in close proximity to the p-loop (purple), which has a known role in determining duty-ratio. Moreover, E466 is directly involved in phosphate coordination in phosphate release[2], which lends support to the DiffNet prediction that changes in this residue are important for determining duty ratio.

Supplementary References

1. Dempster AP, Laird NM, Rubin DB. Maximum Likelihood from Incomplete Data Via the EM Algorithm . *J R Stat Soc Ser B*. 1977. doi:10.1111/j.2517-6161.1977.tb01600.x
2. Llinas P, Isabet T, Song L, et al. How Actin Initiates the Motor Activity of Myosin. *Dev Cell*. 2015. doi:10.1016/j.devcel.2015.03.025
