# Supplementary material for: Deep learning the structural determinants of protein biochemical properties by comparing structural ensembles with DiffNets
Source: Nat Commun. 2021 May 21;12:3023. doi: 10.1038/s41467-021-23246-1 (PMC8140102; doi:10.1038/s41467-021-23246-1)
Supplement: Supplementary file 3 — Reporting Summary [file 41467_2021_23246_MOESM3_ESM.pdf]

nature research

Corresponding author(s): Gregory R Bowman

Last updated by author(s): Apr 2, 2021

Reporting Summary

Nature Research wishes to improve the reproducibility of the work that we publish. This form provides structure for consistency and transparency in reporting. For further information on Nature Research policies, see our Editorial Policies and the Editorial Policy Checklist.

Statistics

For all statistical analyses, confirm that the following items are present in the figure legend, table legend, main text, or Methods section.

n/a	Confirmed	
☐	☒	The exact sample size (*n*) for each experimental group/condition, given as a discrete number and unit of measurement
☐	☒	A statement on whether measurements were taken from distinct samples or whether the same sample was measured repeatedly
☒	☐	The statistical test(s) used AND whether they are one- or two-sided<br>*Only common tests should be described solely by name; describe more complex techniques in the Methods section.*
☒	☐	A description of all covariates tested
☒	☐	A description of any assumptions or corrections, such as tests of normality and adjustment for multiple comparisons
☐	☒	A full description of the statistical parameters including central tendency (e.g. means) or other basic estimates (e.g. regression coefficient) AND variation (e.g. standard deviation) or associated estimates of uncertainty (e.g. confidence intervals)
☒	☐	For null hypothesis testing, the test statistic (e.g. *F*, *t*, *r*) with confidence intervals, effect sizes, degrees of freedom and *P* value noted<br>*Give P values as exact values whenever suitable.*
☒	☐	For Bayesian analysis, information on the choice of priors and Markov chain Monte Carlo settings
☒	☐	For hierarchical and complex designs, identification of the appropriate level for tests and full reporting of outcomes
☒	☐	Estimates of effect sizes (e.g. Cohen's *d*, Pearson's *r*), indicating how they were calculated

Our web collection on statistics for biologists contains articles on many of the points above.

Software and code

Policy information about availability of computer code

Data collection	All molecular dynamics simulation data were generated with Gromacs 5.1.1.
Data analysis	Data analysis was performed with the method developed in the manuscript (DiffNets), which relies on open-source python libraries including mdtraj, scipy, cython, enspara, numpy, matplotlib, click, and pytorch.

For manuscripts utilizing custom algorithms or software that are central to the research but not yet described in published literature, software must be made available to editors and reviewers. We strongly encourage code deposition in a community repository (e.g. GitHub). See the Nature Research guidelines for submitting code & software for further information.

Data

Policy information about availability of data

All manuscripts must include a data availability statement. This statement should provide the following information, where applicable:
- Accession codes, unique identifiers, or web links for publicly available datasets
- A list of figures that have associated raw data
- A description of any restrictions on data availability

The datasets generated during and/or analyzed during the current study are available from the corresponding author on reasonable request.

Field-specific reporting

Life sciences study design

All studies must disclose on these points even when the disclosure is negative.

Sample size	For beta-lacatamase, we used 6.5 microseconds of simulation data for each variant (WT, M182T, M182S, M182V) as this was what was used in a previous study. In total, this is 1,300,420 data points (simulation frames). In the first two experiments (measuring autoencoder reconstruction error and measuring helix 9 classification performance) we curated a dataset of 178,402 data point from WT and M182T simulations. Concretely, we found all simulation frames where helix 9 was compact (described in Methods of paper -- 89,201 data points), and then randomly sampled 89,201 other data points where helix 9 was extended. This sample size proved to be sufficient to train our neural network both to reconstruct protein structures and to classify helix 9 as compact/extended as shown in figures 3 and 4, respectively. For the next section, where we trained with expectation maximization and without a priori knowledge of helix 9, we used all 1,300,420 simulation frames. Based on the success in the previous tasks with less data, this seemed to be a sufficient sample size.<br><br>For the myosin experiments, our raw data contained ~200,000,000 data points. We sub-sampled this data by a factor of 10 to be able to featurize and train the data with a reasonable amount of compute resources.
Data exclusions	For the myosin experiments, our raw data contained ~200,000,000 data points. We sub-sampled this data by a factor of 10 to be able to featurize and train the data with a reasonable amount of compute resources.
Replication	For the reconstruction experiment, we trained the DiffNet with varying numbers of latent variables and demonstrated that it is robust across different choices of latent variable size.<br><br>For the classification experiment, we evaluated helix 9 classification using 5-fold cross validation. This is a standard procedure in machine learning to estimate the accuracy of a model, by evaluating its performance on different slices of the data.<br><br>For experiments with expectation maximization we provide sample means and the associated standard error of the mean for the relevant plots. We also performed measurements across a wide variety of expectation maximization bounds.
Randomization	For the classification experiment, we evaluated helix 9 classification using 5-fold cross validation. This is a standard procedure in machine learning to estimate the accuracy of a model, by evaluating its performance on randomly selected slices of the data.
Blinding	Not applicable.

Reporting for specific materials, systems and methods

We require information from authors about some types of materials, experimental systems and methods used in many studies. Here, indicate whether each material, system or method listed is relevant to your study. If you are not sure if a list item applies to your research, read the appropriate section before selecting a response.

Materials & experimental systems

n/a	Involved in the study
☒	☐ Antibodies
☒	☐ Eukaryotic cell lines
☒	☐ Palaeontology and archaeology
☒	☐ Animals and other organisms
☒	☐ Human research participants
☒	☐ Clinical data
☒	☐ Dual use research of concern

Methods

n/a	Involved in the study
☒	☐ ChIP-seq
☒	☐ Flow cytometry
☒	☐ MRI-based neuroimaging
